# Can donepezil facilitate weaning from mechanical ventilation in difficult to wean patients? An interventional pilot study

**DOI:** 10.1186/s40199-015-0103-z

**Published:** 2015-03-01

**Authors:** Saeed Abbasi, Shadi Farsaei, Kamran Fazel, Samad EJ Golzari, Ata Mahmoodpoor

**Affiliations:** Anesthesiology and Critical Care Research Center, Isfahan University of Medical Sciences, Isfahan, Iran; Department of Clinical Pharmacy and Pharmacy Practice, Isfahan University of Medical Sciences, Isfahan, Iran; Department of Anesthesia and Critical Care Medicine, Bagiatalla University of Medical Sciences, Tehran, Iran; Medical Education Research Center, Tabriz University of Medical Sciences, Tabriz, Iran; Cardiovascular Research Center, Tabriz University of Medical Sciences, Tabriz, Iran

## Abstract

**Background:**

Management of difficult to wean patients is a dilemma for health care system. Recently published studies demonstrated efficacy of donepezil to counteract respiratory depression in sleep apnea. However, to the best of our knowledge, pharmaceutical interventions with donepezil to facilitate weaning have not been tested so far. Therefore in the present study, we evaluated the efficacy of using donepezil on weaning course in difficult to wean patients.

**Methods:**

In this non-randomized interventional clinical study, difficult to wean patients with prior inappropriately depressed respiratory responses were included from two referral intensive care units (ICU) in Iran. Patients with another potentially reasons of weaning failure were excluded from the study. Donepezil was started for eligible patients at dose of 10 mg daily for 2–4 weeks. For the primary outcomes, arterial blood gas (ABG) parameters were also measured before and after intervention to evaluate the possible effects of donepezil on them. In addition, weaning outcomes of patients were reported as final outcome in response to this intervention.

**Results:**

Twelve out of 16 studied patients experienced successful results to facilitate weaning with donepezil intervention. The mean duration of donepezil treatment until outcome measurement was 12 days. There were not any significant differences in ABG parameters among patients with successful and failed weaning trial on day of donepezil initiation. However after donepezil intervention, mean of PCO2 and HCO3 decreased in patients with successful weaning trial and mean of PCO2 increased in those with weaning failure.

**Conclusions:**

Reduced central respiratory drive was infrequently reason of failed weaning attempts but it must be considered especially in patients with hypercapnia secondary to inefficient gas exchange and slow breathing. Our results in the clinical setting suggest that, the use of donepezil can expedite weaning presumably by stimulation of respiratory center and obviate the need to re-intubation in cases of respiratory drive problem in difficult to wean patients. We suggest decrease PCO2 and HCO3 during donepezil steady could be valuable predictors for positive response to donepezil intervention.

## Background

Difficult weaning is a common problem of patients in whom weaning trials were attempted [1]. Since, 26% of medical and surgical patients were considered difficult-to-wean in a prospective cohort study [2]. Similarly, another prospective cohort study found that the incidence of difficult weaning was 39% among patients who needed mechanical ventilaion for more than 12 hours [3].

Both prolonged weaning and duration of mechanical ventilation are associated with increased risk of mortality. Therefore, difficult weaning is an important challenge for critically ill patients with mechanical ventilation support [4]. Management of patients who are difficult-to-wean is based on identification and correction of potential causes related to ventilator dependency [5].

Although impaired respiratory drive is an uncommon cause of weaning failure, but its important role should be highlighted if any definite reason is not found for weaning failure [1]. Therefore, treatment targeting the respiratory drive can be helpful in facilitating weaning of difficult-to-wean patients [6]. Respiratory stimulants such as caffeine, aminophylline and doxapram have been used effectively to increase central respiratory drive and subsequently weaning from mechanical ventilation and avoiding post-extubation apnea in preterm infants [7-9]. In addition, some studies showed beneficial effects of cholinergic drugs like donepezil to stimulate respiratory drive [10,7].

Some advances in understanding the role of central cholinergic modulation in the process of respiration provide a pharmacological basis for explaining beneficial effects of cholinergic drugs as therapeutic agents for disorders related to neural control of breathing [10]. Donepezil is cholinergic drug which reversibly and noncompetitively inhibits centrally-active acetyl cholinesterase [11]. It was first introduced for Alzheimer treatment but many studies showed efficacy and safety of donepezil for different clinical problems such as cognitive impairment resulting from severe traumatic brain injury [12-14]. Nausea, diarrhea, insomnia and infection were reported the most prevalent adverse reactions of donepezil with prevalence of less than 20% in high doses of donepezil [15].

Moreover, findings of conducted animal studies indicated the beneficial effect of systemically administered donepezil to counteract respiratory depression in anesthetized rabbits [16,17]. Recently published clinical studies showed donepezil may improve sleep apnea [18,19]. As a result, it seems donepezil is safe and may be effective medication to stimulate respiratory drive. To the best of our knowledge, pharmaceutical interventions with donepezil in difficult-to-wean patients have not been tested so far. Therefore, in the present study we hypothesized that using donepezil could facilitate weaning course in difficult-to-wean patients.

## Methods

This is an interventional non-randomized clinical study approved by medical ethics committee of Isfahan University of Medical Sciences (Code Number: 292232) and conducted in the intensive care units (ICUs) of two referral hospitals in Iran (Alzahra and Imam reza hospitals).

Intubated patients who failed the first spontaneous breathing trial (SBT) on weaning trial and required up to three SBTs or 7 days to pass a SBT were defined as difficult-to-wean. Difficult-to-wean patients with prior inappropriately depressed respiratory responses when removed from the ventilator were considered eligible to enter in this study.

According to clinical judgment of intensivist, when other possible reasons of weaning failure such as electrolytes and acid–base disorders were ruled out, depressed respiratory response could be considered the cause of this failure.

Since electrolytes and acid–base imbalances (in particular metabolic alkalosis from volume depletion which promoted hypoventilation) could interfere in weaning process, we did our best to control and maintain normal ranges of serum electrolytes and nutritional support in this study and preserve the pH less than 7.45 during the time of active weaning [20].

Patients with unconsciousness and need for sedation or hypothyroidism were excluded from this study.

A continuous electrocardiograms (ECG), heart rate, mean arterial blood pressure, and oxygen saturation were also monitored during SBT. Therefore, patients with weaning-related myocardial ischemia or hemodynamic instability were excluded from the study to omit the potentially negative effects of these confounding factors on weaning failure [21]. Treatment with other potentially respiratory stimulants such as doxapram and medroxyprogesterone was additional criterion to exclude patients from this study.

Donepezil was started for eligible patients according to inclusion and exclusion criteria at dose of 10 mg daily. Because time to steady state for donepezil was expected 15 days [22], donepezil administration was continued at least for 2 weeks and patients were followed up daily during this period and evaluated for readiness of weaning and other endpoints of the study. If weaning was failed during first two weeks, patients would be assessed again for another potential barrier to extubation. In cases with strong clinical suspicion of central respiratory drive problem during weaning, donepezil was continued for another two weeks until 4-week duration of donepezil treatment [23].

For the primary outcomes of the study, oxygenation variables such as partial pressures of oxygen (PO2) and carbon dioxide (PCO2), pH and hemoglobin oxygen saturation (SO2) were measured on day of donepezil initiation and on day of outcome evaluation. Moreover, weaning outcomes of patients were reported as final outcome in response to this intervention. Thereafter for statistical analysis patients were categorized in two groups: patients with successful or failure weaning and then oxygenation variables mentioned above were compared between two groups.

Statistical analysis: Descriptive and statistical analyses were performed. The distribution of continuous variables was assessed by the Kolmogrov-Smirnov test, and continuous data were expressed as mean ± SD. The Mann–Whitney U test and independent sample t-tests were used to assess differences in quantitative data of patients’ characteristics for nonparametric and parametric variables, respectively. For categorical data expressed as percentage, chi-squared test was applied.

## Results

**Figure 1:**
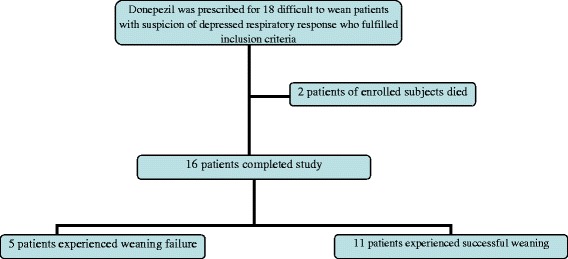
**Flow diagram of the patients received donepezil during study follow up.** Eleven out of 16 difficult to wean patients who completed the study experienced successful weaning in our study.

Eighteen difficult-to-wean patients with suspicion of depressed respiratory response (62 ± 18 years old) were included in this study and finally 16 patients completed the study for outcome measurement. Two patients of enrolled subjects died before any evaluations, therefore data were not applicable to evaluate weaning outcome of them (Figure 1). Characteristics of the patients were summarized in Table 1.

**Table 1:** **Demographic and clinical characteristics of the patients with donepezil intervention to facilitate weaning**

**Patient**	**Age**	**Sex**	**Diagnosis**	**Number of failed weaning trial** ^**α**^	**Duration of MV** ^**α**^	Days of donepezil treatment*	**Outcome of weaning**	**ICU outcome**
**1**	73	M	COPD exacerbation, pneumonia, pulmonary edema	2	10	28	S but re-intubated	D
**2**	26	M	Opioid intoxiction, seizure	2	30^#^	6	S	A
**3**	55	M	COPD exacerbation, PTE, pneumonia	2	5	4	S	A
**4**	73	M	COPD exacerbation, Pneumonia	2	5	8	S	D
**5**	72	M	Brain tumor, PTE, pneumonia	2	10	15	F'	D
**6**	64	F	Abdominoplasty, PTE	2	19	9	S	A
**7**	80	F	PTE, dyspnea, pneumonia, pulmonary edema	1	18	8	S	A
**8**	68	M	Arrest, pneumonia	3	60^#^	6	S	A
**9**	41	F	Arnoldikiary, pneumonia	2	25^#^	13	S	A
**10**	81	F	cholecystectomy	2	32^#^	10	S	A
**11**	78	M	COPD exacerbation, pneumonia	3	33^#^	17	F'	A
**12**	68	M	CVA, pneumonia	3	65^#^	27	S	A
**13**	67	M	PTE, dyspnea, pneumonia	3	60^#^	4	S	A
**14**	81	M	Massive pleural effusion, pneumonia, pulmonary edema	3	57^#^	15	F'	A
**15**	24	M	Multiple trauma, pneumonia	1	57^#^	21	S	A
**16**	46	M	Cerebral tumor, pneumonia, PTE	1	33^#^	15	F'	A

^α^Before donepezil initiation.

^*^From initial donepezil treatment until outcome measurement.

^#^Tracheostomy tube used for mechanical ventilation.

A, alive; COPD, chronic obstructive pulmonary disease; CVA, cerebrovascular accident; D, died; F, female; F', failure; MV, mechanical ventilation; M, male; NA, not applicable and excluded; PTE, pulmonary thromboembolism; S, success.

The study patients had been on mechanical ventilation for 32 ± 21 days prior to study enrollment. In addition, more than 80% of patients had been failed weaning trial more than 2 times before enter to study. Among 16 studied patients, 13 patients had pneumonia, whereas; 30% of them experienced concomitant acute exacerbation of COPD. Pulmonary edema was also reported in 3 patients (Table 1).

The results showed 12 out of 16 patients experienced successful weaning during donepezil intervention. However, in one of the successful weaning trial, re-intubation was occurred during 24 hours and therefore considered as final unsuccessful weaning outcome.

Therefore, 11 patients were categorized as successful weaning and remained patients were classified in unsuccessful weaning group. In comparison of these two groups of patients (successful or failure weaning), there were not any significant differences in age, duration of mechanical ventilation and number of failed weaning trial before donepezil initiation (p-value > 0.05) (Table 2).

**Table 2:** **Comparison of clinical parameters in patients with successful and unsuccessful weaning trial**

**Parameters**	**Successful weaning**	**Unsuccessful weaning**	p-value *
	**(N = 11)**	**(N = 5)**	
Age (Mean ± SD)	58.8 ± 20.1	70.0 ± 14.0	0.22
duration of mechanical ventilation (Mean ± SD)	34.2 ± 22.6	28.6 ± 19.6	0.83
duration of donepezil treatment (Mean ± SD)	10.6 ± 7.3	18.0 ± 5.7	0.03

*Mann–Whitney U test was performed.

**Figure 2:**
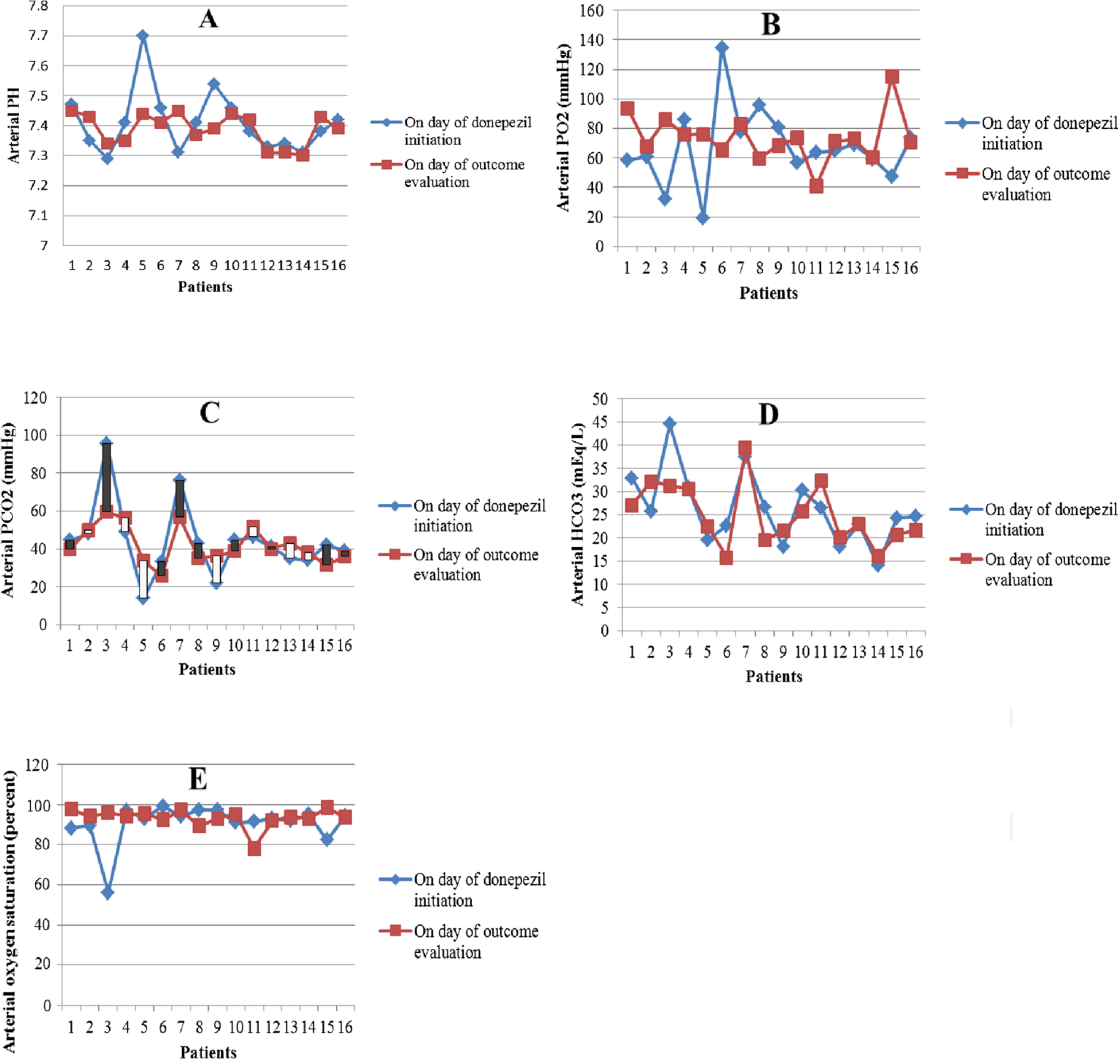
**Variation of Arterial blood gas during donepezil treatment.** Variations in arterial PH, PO2, PCO2, HCO3 and oxygen saturation were demonstrated in part **A**, **B**, **C**, **D** and **E** respectively. For each patient the first reported parameter was related to donepezil initiation day and the last one demonstrated that parameter on weaning day or until donepezil treatment was continued in cases of weaning failure. Patients with number 5, 11, 14 and 16 had unsuccessful attempts for weaning.

The effects of donepezil on arterial blood gas (ABG) were also evaluated on day of donepezil initiation and on day of outcome measurement. Variations in arterial PH, PO2, PCO2, HCO3 and oxygen saturation were demonstrated in part A, B, C, D and E of Figure 2 respectively. Results showed patients had no evidence of primary metabolic alkalosis at weaning time.

According to independent sample t-test, there were not any significant differences in ABG parameters between successful and unsuccessful weaning patients on day of donepezil initiation (p-value > 0.05). In patients with successful weaning trial, paired sample analyses revealed mean of PCO2 and HCO3 decreased, whereas mean of PO2 and PO2 saturation increased after donepezil intervention. Although, in those with failed weaning, mean of PCO2 increased after donepezil intervention. However none of these statistical analyses were significant (Table 3).

**Table 3:** **Arterial blood gas parameters before and after donepezil intervention**

	**ABG parameters**	**Group**	**Mean ± SD**	**p-value**
Successful weaning (N = 11)	PH	Before	7.39 ± .076	0.96
		After	7.38 ± .047	
	PO2	Before	73.2 ± 27.1	0.84
		After	75.5 ± 15.3	
	PCO2	Before	48.2 ± 20.6	0.23
		After	42.8 ± 11.3	
	HCO3	Before	27.4 ± 8.0	0.19
		After	25.1 ± 7.4	
	PO2 saturation	Before	89.9 ± 12.1	0.29
		After	94.49 ± 2.4	
Unsuccessful weaning or re-intubation (N = 5)	PH	Before	7.46 ± 0.15	0.19
		After	7.40 ± 0.06	
	PO2	Before	54.7 ± 20.9	0.67
		After	68.2 ± 19.6	
	PCO2	Before	35.5 ± 13.0	0.192
		After	39.8 ± 7.0	
	HCO3	Before	23.5 ± 7.1	0.132
		After	23.9 ± 6.1	
	PO2 saturation	Before	92.4 ± 2.7	0.99
		After	91.7 ± 7.8	

PCO2: carbon dioxide, PO2: Partial pressures of oxygen, SO2: hemoglobin oxygen saturation and SD: standard deviation.

## Discussion

This present study was performed in difficult-to-wean patients with potential respiratory drive problems which caused repeatedly failing spontaneous breathing attempts. Donepezil administration in our study enabled successful SBT and weaning in nearly 70% of patients. Our patients manifested hypercapnia and hypopnea when removed from the ventilator in prior weaning trials which promoted the probability of reduced ventilator central drive before donepezil initiation. Since, we initially could find neither reversible causes for repeated failure to prior weaning trials, nor any contraindication for the use of donepezil, we empirically used donepezil to facilitate weaning in these patients.

According to recent systematic review and meta-analysis, 10 mg donepezil was well tolerated without any life threatening effect [24]. In addition, regarding to the long half-life of donepezil (70 hours) it is unlikely that adverse reactions of donepezil occurs in first days of its initiation [25]. Therefore, deaths of two patients in the initial days of donepezil intervention might be related to poor clinical condition of them not donepezil adverse reactions. Also it should be mentioned that no related adverse reaction of donepezil was reported during follow up of our study.

Etiologies of failures upon removal from the ventilator in spite of donepezil treatment in our study were not readily clear. Although it seems patients apparently had a depressed ventilatory drive initially, but possible inadequate resolution of the illness that had caused mechanical ventilation and/or progress of new problem could incorporate to weaning failure in few patients.

Muscle fatigue is one of the frequent causes of weaning failure manifested ultimately by rising in PCO2 [26]. Weaning failure in our study might be contributed to muscle fatigue during donepezil intervention. Because as mentioned in results, mean PCO2 rose in patients with unsuccessful weaning in this period. If fatigue developed during donepezil treatment, further stimulation of the respiratory muscles with donepezil would have inevitably made the fatigue worse and patients would have weaning failure or required re-intubation.

In addition, in a patient with cervical tumor, phrenic nerve was involved and patient had also peripheral neuropathy. Since, diaphragm muscle is primarily innervated by the phrenic nerve, partial damage of that in surgical removal of cervical tumor could be a reason for failed response to donepezil intervention. Moreover, metabolic alkalosis may be another causative factor for re-intubation of the first patient in our study because compensatory hypoventilatory response to metabolic alkalosis disposed patient to weaning failure or re-intubation [27]. Some studies maintained pH less than 7.4 during the time of active weaning [28].

Most of patients had history of pneumonia in our study which justified extubation failure and predisposition of them in difficult-to-wean condition. Pneumonia at the initiation of ventilation was one of the best predictors of extubation failure for patients following a successful SBT [29].

Cholinergic stimulation to develop respiratory coordination [30], increasing neuromuscular transmission and improving the effectiveness of upper airways muscles are some reasons which donepezil can accelerate weaning [31]. Not only cholinergic drugs can increase chemoreflex response to hypoxia by acting upon carotid body and stimulate respiratory center, but also play an important role in the neural control of respiration [31-33]. In addition, beneficial effects of cholinergic drugs to increase saliva secretion can reduce collapsibility of upper airways and contribute in this result [34]. Although different mechanisms can explain the beneficial effect of donepezil to expedite weaning but the exact mechanism is not clear.

By considering these mechanisms, donepezil facilitated the weaning of most patients in our study. We conclude that ABG parameters can be valuable factors to predict patient response to donepezil intervention. As our results also showed, decrease of PCO2 and HCO3 during donepezil steady state would give us good news about positive response of donepezil and possibly successful weaning trial in near future. Conversely, increase or unchanged PCO2 during steady state of donepezil could promote clinicians to seek for other reasons of weaning failure such as muscle fatigue. However, because of small sample size analyzed in this study differences in ABG parameters between two groups (successful and failure weaning) were not statistically significant.

Various pharmacokinetic parameters in population study and contribution of different factors in weaning process might be the reasons of diverse time needed for donepezil treatment to achieve its response goal during the study. As expected, mean duration of donepezil until successful weaning was 12 days which was parallel to the time required for donepezil to receive its steady state [20]. However, clinical benefit of donepezil may occur before or after steady state of donepezil. Therefore we suggest if weaning is not facilitated during 2 weeks of donepezil initiation and clinician do not find any other factor for this failure, patient may benefit from 4 weeks therapy. This study protocol justify the significant higher duration of donepezil treatment in patients who failed weaning trial compared to those with successful weaning outcomes.

Finally, potential limitations of the present study should be mentioned. First, however patients ruled out for other potential barriers of weaning in our study but other reasons of weaning failure might not be completely resolved or some new barriers evolved during the study. So, outcome of weaning failure in our population is not necessarily or primarily related to respiratory drive problem which is discussed in patients with unsuccessful weaning trial. Another limitation which should be pointed out is that relatively small number of studied patients. Despite more than 1 year study duration and inclusion of consecutive patients in two centers; only limited number of patients entered the study according to inclusion and exclusion criteria. A non-randomized design of this study without control group is the third limitation. Therefore, these results require further confirmation with larger controlled clinical trials to clarify and establish donepezil role in the management of difficult-to-wean patients. Additional studies should be conducted to compare the efficacy of donepezil with other respiratory center stimulants such as doxapram or cholinergic drugs to facilitate weaning in difficult-to-wean patients with respiratory drive problem. Moreover, the effect of donepezil on weaning of patients with different diagnoses can be evaluated in future studies.

## Conclusion

According to safety consideration and previous study in obstructive sleep apnea (OSA) we evaluated 10 mg of donepezil during 2–4 weeks in our study [19]. In accordance with our findings, donepezil also supported breathing regulation in OSA patients [19]. To the best of our knowledge, this is the first study in which donepezil was given as respiratory drive stimulant in difficult-to-wean patients in order to expedite the weaning.

However ventilator drive failure uncommonly exists on the list of reversible causes of weaning problem, but it may prolong mechanical ventilation and lead impaired weaning. We think this condition may be overlooked by the other factors which contribute to weaning failure and may remain unrecognized. Our results showed donepezil could be considered as useful treatment option in this condition.
